# Supplementary material for: Rationally-engineered reproductive barriers using CRISPR & CRISPRa: an evaluation of the synthetic species concept in Drosophila melanogaster
Source: Sci Rep. 2018 Sep 3;8:13125. doi: 10.1038/s41598-018-31433-2 (PMC6120925; doi:10.1038/s41598-018-31433-2)

Supplementary Information

Rationally-engineered reproductive barriers using CRISPR & CRISPRa: an evaluation of the synthetic species concept in *Drosophila melanogaster*

Andrew J Waters[1], Paolo Capriotti[1], David C. A. Gaboriau[2], Philippos Aris Papathanos[3] & Nikolai Windbichler[1]

[1]*Department of Life Sciences, Sir Alexander Fleming Building, Imperial College London, South Kensington, London, SW7 2AZ, U.K.*

[2]*Facility for Imaging by Light Microscopy, NHLI, Imperial College London, South Kensington, London, SW7 2AZ, U.K.*

[3]*Section of Genomics and Genetics, Department of Experimental Medicine, University of Perugia, 06132 Perugia, Italy.*

Supplementary Figure 1. (A) Protein alignment between the histone acetyltransferase orthologues P300 (*Homo sapiens*) and Nejire (*Drosophila melanogaster*). The previously identified core region of P300 aligns with high identity to the putative core region of Nejire; Bromo Domain (BD), RING domain and Histone Acetyltransferase (HAT) domain have high % identity, the HAT aspartate catalytic residue D1399 in P300 corresponds to D2046 in Nejire Core[26]. **(B)** dCas9-VPR, dCas9-P300 Core and dCas9-Nejire Core protein fusion schematics; FLAG epitope (DYKDDDDK) for antibody detection, Nuclear Localization Sequence (NLS). **(C)** Aberrant morphology of eyes seen with dCas9-Nejire Core in presence of various gene-specific sgRNAs. **(D)** dCas9-VPR expressed in eyes with sgRNAs show no aberrant morphology, except in combination with *eve*-sgRNA. **(E)** dCas9-P300 Core gives no observable aberrant morphology when expressed in eyes with sgRNAs.

Supplementary Figure 2. Plasmid maps for cloning and expression vectors used.

Supplementary Table 1. Statistical analysis of INDEL inheritance as a measure of fitness. Equal numbers of Heterozygous balanced mutants were crossed as virgins and presence and absence of balancer scored in F1 generation. Expected frequency for homozygous F1 individuals if no fitness cost present would be 1/3 of total counted. X^2 shows the calculated Chi-Squared value calculated from Observed and Expected Frequencies of the two genotypic classes for each INDEL. With 1 degree of Freedom of used X^2 values higher than 6.63 (p=0.01) indicate a significant difference between observed and expected frequency of homozygous mutants in the F1 generation. This significant difference is mostly significantly fewer than expected when there is a difference, however engrailed-5/17/99 gives significantly more homozygous mutants than expected in the F1 indicating that there is less fitness costs than the balancer for this line.

Supplementary Table 2. Transgenic lines crossed as females to tester stocks to determine lethality. Compare with Figure 6A. Female virgin transgenic stocks crossed to male tester stocks. Associated % Lethality is similar to that seen for lines crossed as males (Figure 6A) to tester stocks.

Supplementary Table 3. P-element insertion loci. P-element flanking sequence derived from Inverse PCR of 5' element, 15 nucleotides of flanking sequence shown each side of insertion. Genomic location of insertion along with strand and nearest 5' and 3' gene are shown. If element inserts within a gene (either intron or UTR) this is denoted by the nearest 5' Gene shown in brackets. Distances shown in kilobases (kb) *np* = not performed.

Supplementary Table 4. Assessment of transgenic fitness costs in homozygous viable lines. Scored embryo number and subsequent scored larval survival numbers are shown for each homozygous strain. Percentage Lethality calculated as Dead Embryo number divided by Total number of embryos.

Supplementary Technical Cross 1: Activation Domain and qRT-PCR sgRNA screen in eyes. Cross of dCas9-Activators expressed in eyes from *GMR*-GAL4 driver in the eyes to sgRNA to assess strength of Activation fusions and sgRNA transactivation potential. In total 13 sgRNAs were crossed to female dCas9-Activator lines and each assessed for activation potential using gene specific primers on cDNA made from head RNA. Control is w1118 flies which contain no sgRNA. (**A**)dCas9-VPR. (**B**) dCas9-p300 Core. (**C**) dCas9-Nejire Core.

Supplementary Technical Cross 2: CRISPRa Lethality Screen: Female activator-GAL4 lines crossed to male sgRNAs. Genotypes scored in F1 differ depending on the balancer genotype of the female line crossed (See Supplementary Data Table 1 for which crosses used which strategy) **(A)** Tubulin-GAL4 driving UAS::dCas9-VPR is balanced over a multiple 2nd and 3rd fused balancer; progeny can inherit either UAS::dCas9-VPR and *αTubulin-84b*-GAL4 or the balancer. sgRNA lines were crossed as males and scored for presence of absence of balancer phenotypes. % Survival was calculated as shown. (**B**) When both UAS::dCas9-VPR and *driver*-GAL4 are balanced, 4 possible phenotypes are scored in the F1. (**C**) UAS::dCas9-VPR is homozygous and *driver*-GAL4 is balanced in female parent (**D**) UAS::dCas9-VPR is heterozygous and *driver*-GAL4 is homozygous in female parent. (**E**) Both UAS::dCas9-VPR and *driver*-GAL4 are homozygous, phenotypes not scored in F1, presence of absence of progeny used to discern lethality.

Supplementary Technical Cross 3: qRT-PCR and Protein Analysis in the embryo (A) *αTubulin-84b*-GAL4 is used to drive UAS::dCas9-VPR and crossed to *eve*-sgRNA (also *hid1*-sgRNA for mRNA analysis) males. Because the *αTubulin-84b*-GAL4 is not viable as a homozygous insertion it was not possible to obtain non-segregating lines. F1 progeny are a mix of two genotypes, one that can putatively activate expression at the *eve* (or *hid*) locus and a balancer genotype. As embryos were assessed it was not possible to distinguish F1 genotypes (dominant marker of the balancer becomes visible at the larval stage onwards). We therefore pooled F1 progeny and compared against the control line which contains no sgRNA in order to discern over-expression phenotypes.

Supplementary Technical Cross 4: CRISPR/Cas9 INDEL Creation. Female *vasa:*Cas9 line crossed to male sgRNAs (A) Strategy to induce INDEL mutations on the 2nd chromosome using a 2nd Chromosome balancer to maintain the putative INDEL through successive generations. **(B)** A more complex strategy is required for establishing chromosome 3 INDEL mutations as both the sgRNA and Cas9 are integrated at the AttP-9A (VK00027) on chromosome 3 which needed to be removed to accurately assess the viability of the mutation. The putative INDEL induced in female germ-cells is selected with GFP in order to easily screen for GFP absence in later generations (sgRNA is marked by Vermillion complementation, which is not possible to score in a *Mini-White* background); a balancer that maintains the mutation on chromosome 3 used. GFP is lost through recombination in the female germ-line in the absence of a balancer in the final step. INDELs were then reconfirmed to be present and maintained with a 3rd chromosome balancer.

Supplementary Technical Cross 5: Synthetic Lethal Element Screen. Testing for lethality involved crossed heterozygous male transgenic lines to w1118 female flies which are wild-type for sgRNA binding sites at the *eve* and *hid* loci. **(A)** Transgenics derived from ΦC31 integration using the AttB/AttP sites **(B)** Single Barrier vector integrated using P-elements at various (unknown) chromosomal locations. **(C)** Double Barrier vectors integrated at various locations with P-elements, integrated into a genome protective at the *eve* and *hid* loci.

Supplementary Technical Cross 6: qRT-PCR Embryo Analysis for P-Element Insertions. (A) Heterozygous male P-element bearing flies were crossed to tester stocks of w1118 (wild-type) or the protective *eve*Δ11 strain. F1 embryos segregating the transgene were collected as a pooled population as heterozygous parent were crossed (homozygotes for the tested transgenic lines were inviable). Embryos were aged, collected and RNA extracted, cDNA synthesized and *eve* expression analysed by qRT-PCR.

Supplementary Figure 1

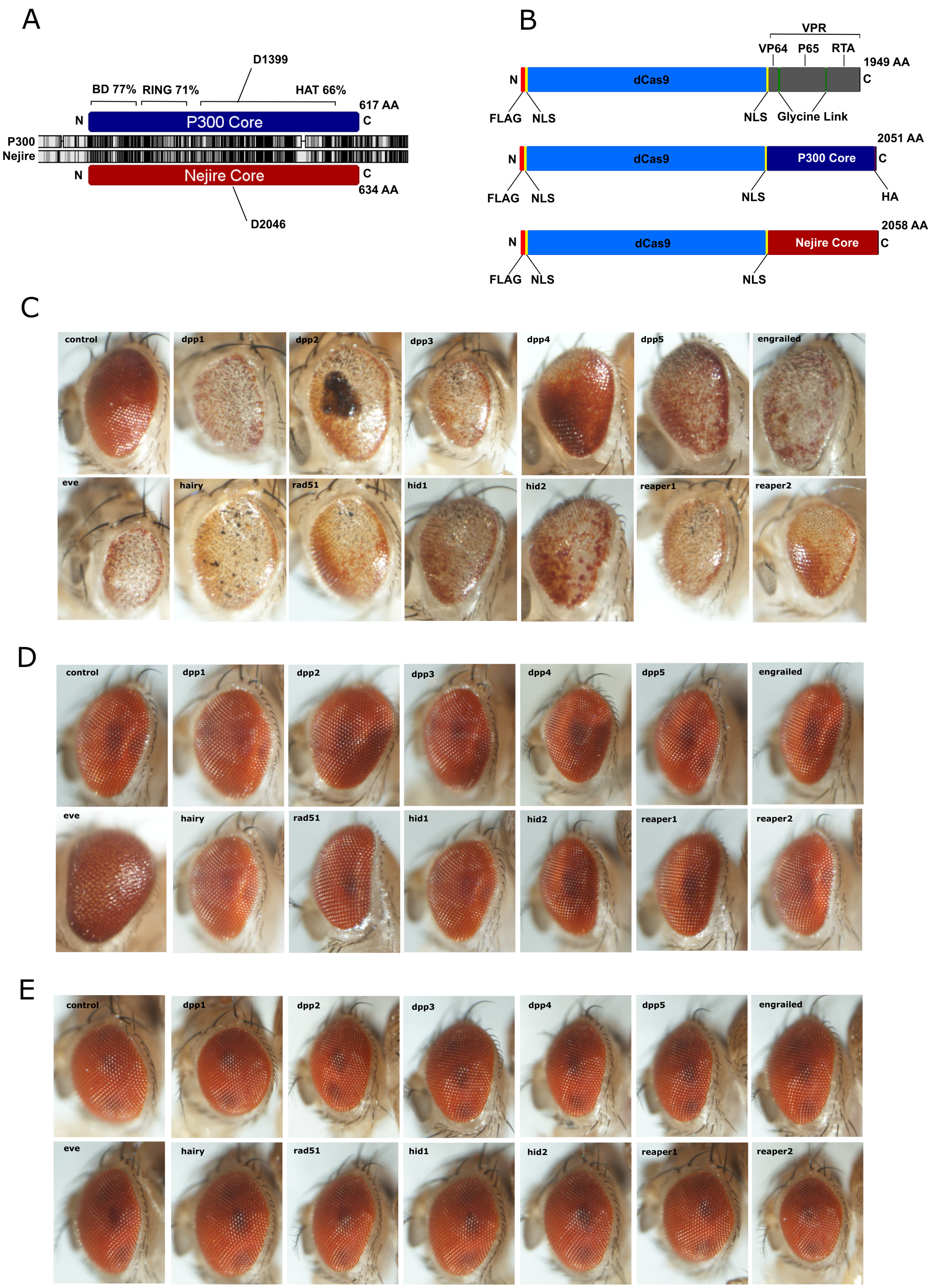

Supplementary Figure 2

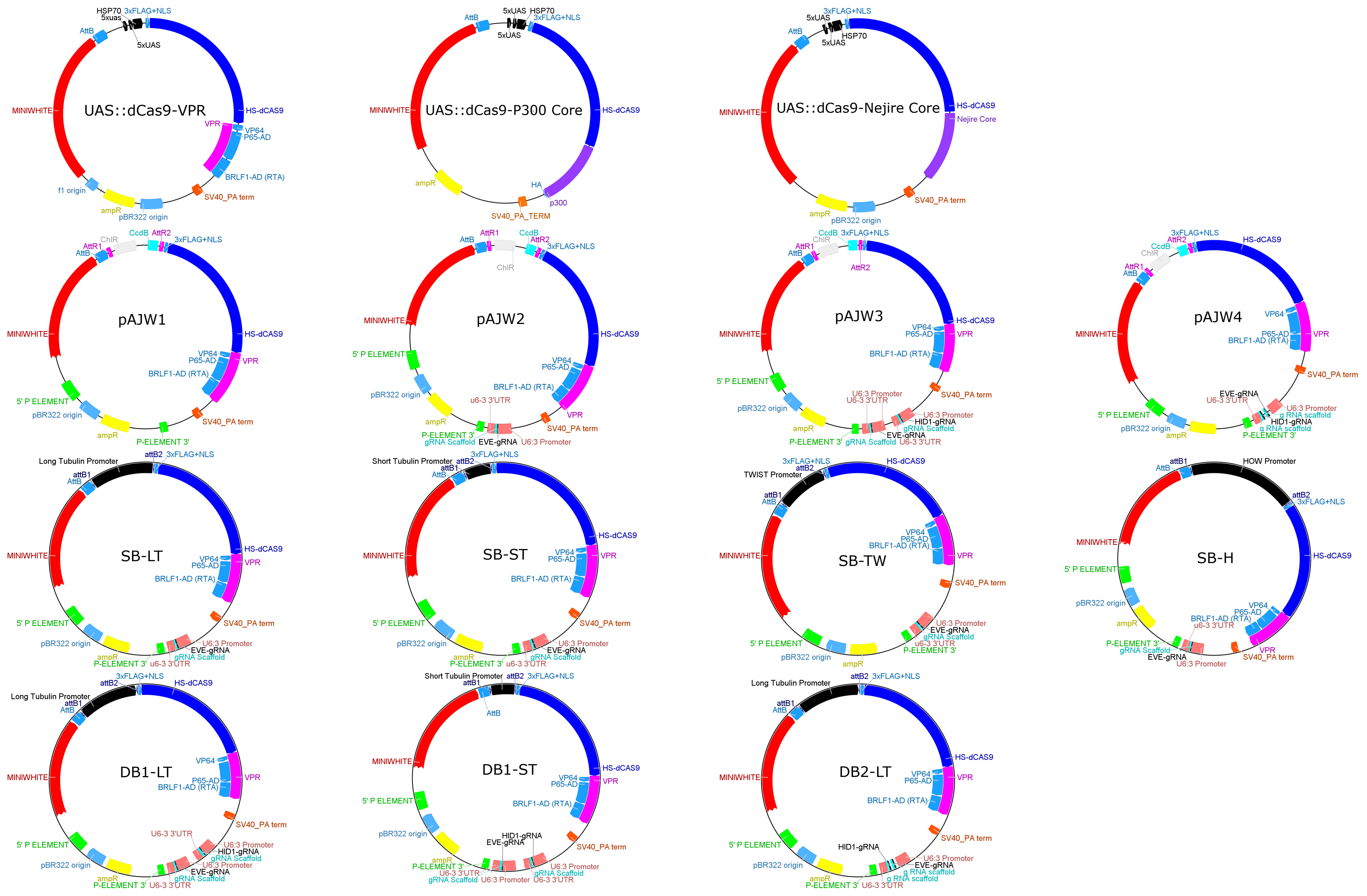

Supplementary Table 1

INDEL Line	Homozyous Δ / Total	Observed Homozyous Δ	Expected Homozyous Δ	X^2 Value	Significantly Fewer Homozyous Δ than expected (P=0.01 with 1 Degree of Freedom)
dpp2-1	38/202	38	67	19.27	Yes
dpp2-4	18/132	18	44	23.05	Yes
dpp2-3/5	92/309	92	103	1.76	No
dpp2-6	31/93	31	31	0.00	No
engrailed-1/2/3	35/102	35	34	0.04	No
engrailed-5/17/99	243/582	243	194	18.56	No (More than expected)
engrailed-15	43/137	43	46	0.24	No
eve-2/3/11	204/587	204	196	0.53	No
eve-12	54/164	54	55	0.02	No
eve-13/14	105/315	105	105	0.00	No
eve-16	31/219	31	73	36.25	Yes
hairy-1	31/97	31	32	0.09	No
hairy-3/10	22/63	22	21	0.07	No
hid1-13	50/147	50	49	0.03	No
hid1-15	35/103	35	34	0.03	No
hid1-19	74/223	74	74	0.01	No
rad51-10	36/112	36	37	0.08	No
rad51-28	25/72	25	24	0.06	No
reaper1-8	80/253	80	84	0.34	No
reaper1-9	0/120	0	40	60.00	Yes
reaper1-10	44/117	44	39	0.96	No

Supplementary Table 2

Transgenic Strain	sgRNA Target	Integration	Homozygous Viable	Tester Strain	Progeny (transgenic / total)	Synthetic Lethality (%)
SB-*H*	*eve*	AttB/AttP	Yes	wild-type	38/74	0.0
SB-*H*	*eve*			*eve* Δ11	44/87	0.0
SB-*LT*	*eve*		No	wild-type	0/227	100.0
SB-*LT*	*eve*			*eve* Δ11	92/185	0.5
SB-*ST*	*eve*		Yes	wild-type	12/51	52.9
SB-*ST*	*eve*			*eve* Δ11	88/173	0.0
SB-*TW*	*eve*		Yes	wild-type	18/63	42.9
SB-*TW*	*eve*			*eve* Δ11	53/106	0.0
SB-*LT* -1-4	*eve*	P-Element	No	wild-type	21/150	72.0
SB-*LT* -1-4	*eve*			*eve* Δ11	59/119	0.8
SB-*LT* -4-2	*eve*		No	wild-type	3/105	94.3
SB-*LT* -4-2	*eve*			*eve* Δ11	85/172	1.2
SB-*LT* -4-3	*eve*		No	wild-type	10/79	74.7
SB-*LT* -4-3	*eve*			*eve* Δ11	31/64	3.1
SB-*LT* -5-1	*eve*		No	wild-type	6/160	92.5
SB-*LT* -5-1	*eve*			*eve* Δ11	40/81	1.2
SB-*LT* -7-1	*eve*		No	wild-type	171/86	0.0
SB-*LT* -7-1	*eve*			*eve* Δ11	100/203	1.5
SB-*LT* -7-2	*eve*		No	wild-type	94/186	0.0
SB-*LT* -7-2	*eve*			*eve* Δ11	90/180	0.0
SB-*LT* -8-1	*eve*		No	wild-type	36/147	51.0
SB-*LT* -8-1	*eve*			*eve* Δ11	73/145	0.0
SB-*LT* -9-1	*eve*		No	wild-type	79/157	0.0
SB-*LT* -9-1	*eve*			*eve* Δ11	82/165	0.6

Supplementary Table 3

Transgenic Strain	5' Gene	Distance to 5' Gene (kb)	3' Gene	Distance to 3' Gene (kb)	Genomic Location of Insertion [strand]	Flanking Sequence (5'-3')
SB-*LT*-1-4	(CG32179)	Inserts in 5'UTR of CG32179 (0.309 from TSS)	CG7484	0.612	3L:17,647,057 [+]	ACCTTGTTTTCTTCT-**P**-ATGCGTTGGTCGATT
SB-*LT*-4-2	*np*	*np*	*np*	*np*	*np*	*np*
SB-*LT*-4-3	CG2530	0.189	CG43131	21.467	3R:5,087,203 [-]	ACTCTGCGCTCGTCT-**P**-CCTGCGACTGTGTGT
SB-*LT*-5-1	*np*	*np*	*np*	*np*	*np*	*np*
SB-*LT*-7-1	(CG7858)	Inserts in 5'UTR of CG7858 (0.20 from TSS)	CG6310	0.679	3L:11,070,545 [-]	AAATTGAGAGTGACG-**P**-ATATGGACGAGCTGC
SB-*LT*-7-2	(CG7858)	Inserts in 5'UTR of CG7858 (0.20 from TSS)	CG6310	0.679	3L:11,070,545 [-]	AAATTGAGAGTGACG-**P**-ATATGGACGAGCTGC
SB-*LT*-8-1	(CG42551)	Inserts in intron of CG42551 (9.312 from TSS)	CG1345	9.981	3R:24,152,818 [-]	GCACGTAGGCCATCC-**P**-GAAGGAGAGCCGAAA
SB-*LT*-9-1	*np*	*np*	*np*	*np*	*np*	*np*
DB1-*LT*-1	CG10038	22.973	CG10013	0.803	3R:12,325,957 [-]	TAGTCTGTCCAACAA-**P**-TGGGGCGCAGACCAT
DB1-*ST*-1	*np*	*np*	*np*	*np*	*np*	*np*
DB2-*LT*-1	*np*	*np*	*np*	*np*	*np*	*np*
DB2-*LT*-2	*np*	*np*	*np*	*np*	*np*	*np*
DB2-*LT*-3	(CG3964)	Inserts in intron of CG3964 (3.099 from TSS)	CR44078	3.657	2L:3,865,493 [+]	GTTATATTTTCGCAG-**P**-TGCTCACTTTTGCAT

Supplementary Table 4

Strain	Total Embryos	Surviving Larvae	Dead Embryos	Lethality (%)
w1118	529	501	43	8.6
eve Δ11	471	443	42	9.5
SB-*TW*	502	451	50	11.1
SB-*ST*	360	302	54	17.9

Supplementary Technical Cross 1: Activation Domain and qRT-PCR sgRNA screen in eyes

A

X^{w1118};UAS::dCas9-VPR[W+];*GMR*-GAL4[W+]
X^{w1118};UAS::dCas9-VPR[W+];*GMR*-GAL4[W+]

X

Sample → Xv^1y^1;-;sgRNA[V+] / Y ;-;sgRNA[V+] → Xv^1y^1/Y;UAS::dCas9-VPR[W+];*GMR*-GAL4[W+] / X^{w1118} ; - ;sgRNA[V+]

Collect Tissue (30 heads)
RNA extraction
cDNA Synthesis
qRT-PCR

Control → X^{w1118};-;- / Y ;-;- → X^{w1118}/Y;UAS::dCas9-VPR[W+];GMR-GAL4[W+] / X^{w1118} ; - ; -

Collect Tissue (30 heads)
RNA extraction
cDNA Synthesis
qRT-PCR

B

X^{w1118};UAS::dCas9-P300 Core[W+];*GMR*-GAL4[W+]
X^{w1118};UAS::dCas9-P300 Core[W+];*GMR*-GAL4[W+]

X

Sample → Xv^1y^1;-;sgRNA[V+] / Y ;-;sgRNA[V+] → Xv^1y^1/Y;UAS::dCas9-p300 Core[W+];*GMR*-GAL4[W+] / X^{w1118} ; - ;sgRNA[V+]

Collect Tissue (30 heads)
RNA extraction
cDNA Synthesis
qRT-PCR

Control → X^{w1118};-;- / Y ;-;- → X^{w1118}/Y;UAS::dCas9-P300 Core[W+];GMR-GAL4[W+] / X^{w1118} ; - ; -

Collect Tissue (30 heads)
RNA extraction
cDNA Synthesis
qRT-PCR

C

X^{w1118};UAS::dCas9-Nejire Core[W+];*GMR*-GAL4[W+]
X^{w1118};UAS::dCas9-Nejire Core[W+];*GMR*-GAL4[W+]

X

Sample → Xv^1y^1;-;sgRNA[V+] / Y ;-;sgRNA[V+] → Xv^1y^1/Y;UAS::dCas9-Nejire Core[W+];*GMR*-GAL4[W+] / X^{w1118} ; - ;sgRNA[V+]

Collect Tissue (30 heads)
RNA extraction
cDNA Synthesis
qRT-PCR

Control → X^{w1118};-;- / Y ;-;- → X^{w1118}/Y;UAS::dCas9-Nejire Core[W+];GMR-GAL4[W+] / X^{w1118} ; - ; -

Collect Tissue (30 heads)
RNA extraction
cDNA Synthesis
qRT-PCR

Supplementary Technical Cross 2: CRISPRa Lethality Screen

A

X^{w1118};UAS::dCas9-VPR[W+];*aTubulin-84b*-GAL4[W+]
X^{w1118}; SM5=TM6b

X

Screen → Xv^1y^1;-;sgRNA[V+] / Y ;-;sgRNA[V+] →

- Xv^1y^1/Y;UAS::dCas9-VPR[W+];*aTubulin-84b*-GAL4[W+] / X^{w1118} ; - ;sgRNA[V+] *
- X^{w1118} ; SM5=TM6b / Xv^1y^1/Y ; - ; sgRNA[V+]

%survival=(((*Non-Balanced)/(Total Flies))X100)X2

Control → X^{w1118};-;- / Y ;-;- →

- X^{w1118} ;UAS::dCas9-VPR[W+];*aTubulin-84b*-GAL4[W+] / X^{w1118}/Y; - ; - *
- X^{w1118} ; SM5=TM6b / X^{w1118}/Y; - ; -

%survival=(((*Non-Balanced)/(Total Flies))X100)X2

B

Screen

X^{w1118};UAS::dCas9-VPR[W+];*driver*-GAL4[W+] / X^{w1118}; Cyo ; TM6b X Xv^1y^1;-;sgRNA[V+] / Y ;-;sgRNA[V+] →

- Xv^1y^1/Y;UAS::dCas9-VPR[W+];*driver*-GAL4[W+] / X^{w1118} ; - ;sgRNA[V+] *
- Xv^1y^1/Y;UAS::dCas9-VPR[W+];TM6b / X^{w1118} ; - ;sgRNA[V+]
- Xv^1y^1/Y; CyO ;*driver*-GAL4[W+] / X^{w1118} ; - ;sgRNA[V+]
- Xv^1y^1/Y; CyO ;TM6b / X^{w1118} ; - ;sgRNA[V+]

%survival=(((*Non-Balanced)/(Total Flies))X100)X4

C

Screen

X^{w1118};UAS::dCas9-VPR[W+];*driver*-GAL4[W+] / X^{w1118};UAS::dCas9-VPR[W+]; TM6b X Xv^1y^1;-;sgRNA[V+] / Y ;-;sgRNA[V+] →

- Xv^1y^1/Y;UAS::dCas9-VPR[W+];*driver*-GAL4[W+] / X^{w1118} ; - ;sgRNA[V+] *
- Xv^1y^1/Y;UAS::dCas9-VPR[W+];TM6b / X^{w1118} ; - ;sgRNA[V+]

%survival=(((*Non-Balanced)/(Total Flies))X100)X2

D

Screen

X^{w1118};UAS::dCas9-VPR[W+];*driver*-GAL4[W+] / X^{w1118}; CyO ;*driver*-GAL4[W+] X Xv^1y^1;-;sgRNA[V+] / Y ;-;sgRNA[V+] →

- Xv^1y^1/Y;UAS::dCas9-VPR[W+];*driver*-GAL4[W+] / X^{w1118} ; - ;sgRNA[V+] *
- Xv^1y^1/Y; CyO ;*driver*-GAL4[W+] / X^{w1118} ; - ;sgRNA[V+]

%survival=(((*Non-Balanced)/(Total Flies))X100)X2

E

Screen

X^{w1118};UAS::dCas9-VPR[W+];*driver*-GAL4[W+] / X^{w1118};UAS::dCas9-VPR[W+];*driver*-GAL4[W+] X Xv^1y^1;-;sgRNA[V+] / Y ;-;sgRNA[V+] →

- Xv^1y^1/Y;UAS::dCas9-VPR[W+];*driver*-GAL4[W+] / X^{w1118} ;UAS::dCas9-VPR[W+] ;sgRNA[V+]

%survival=Viable or Lethal

Non-standard Nomenclature:
A/B = either A or B, not both genotypes
→ Next generation
⋯→ Taken Forward/Crossed to
Recombination Event
?Δ Putative INDEL mutation
Δ Confirmed INDEL mutation
?{P}? P Element Insertion with unknown insertion point

Supplementary Technical Cross 3: qRT-PCR and Protein Analysis in the embryo

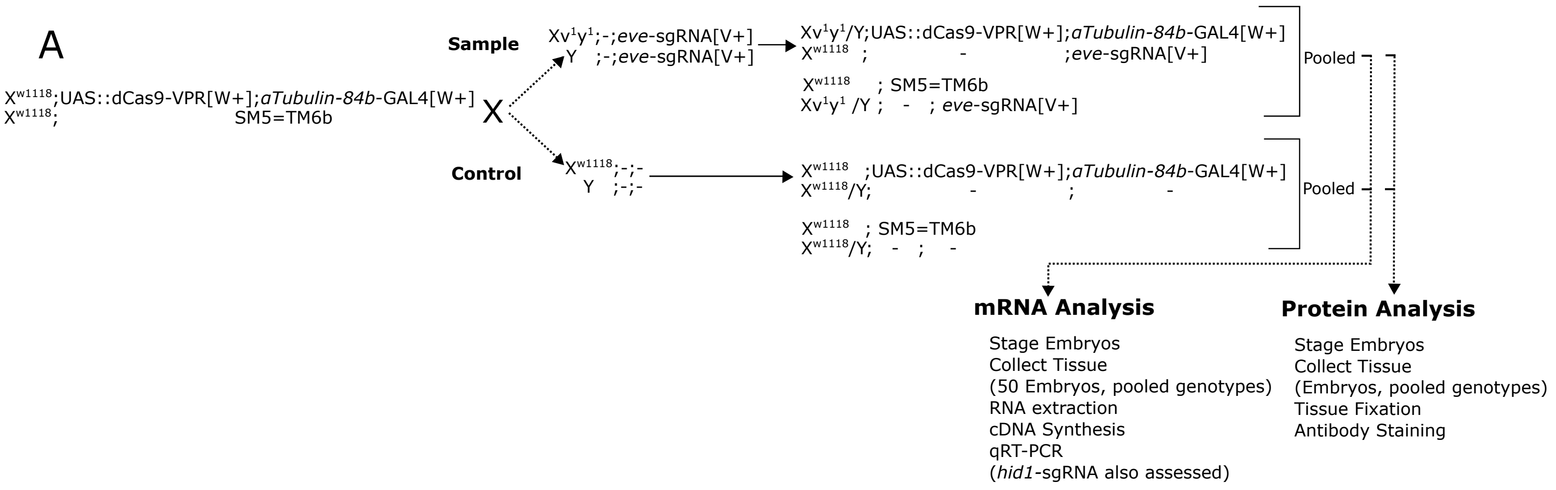

Supplementary Technical Cross 4: CRISPR/Cas9 INDEL Creation

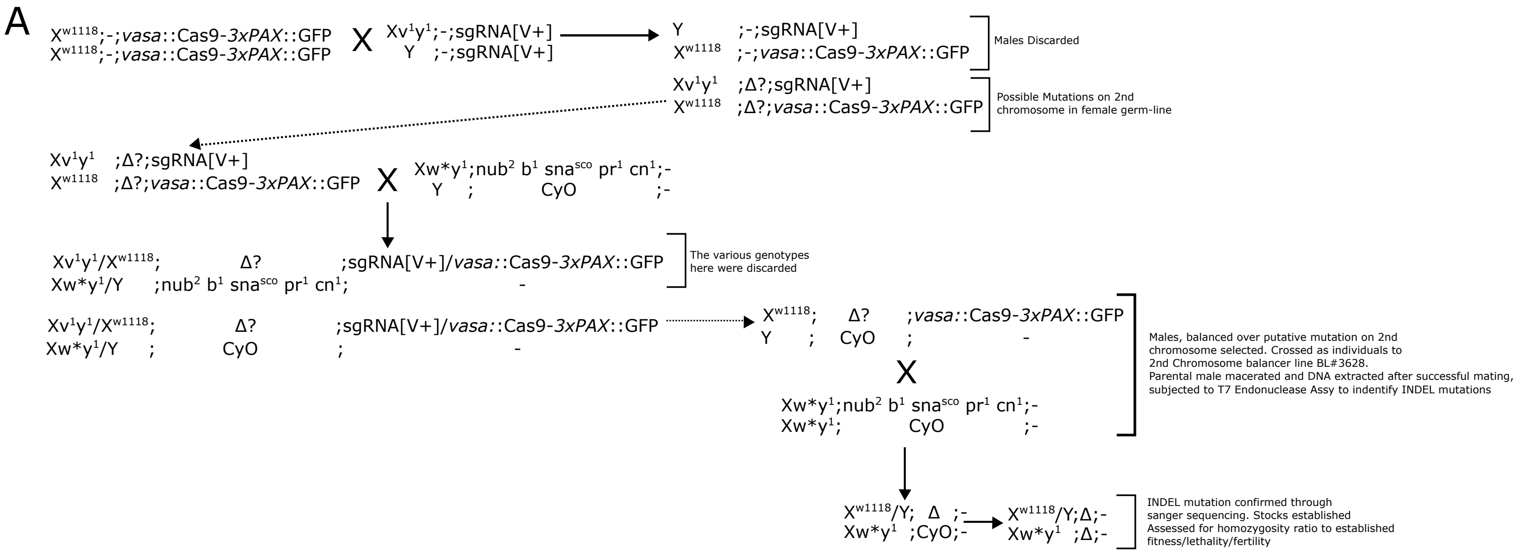

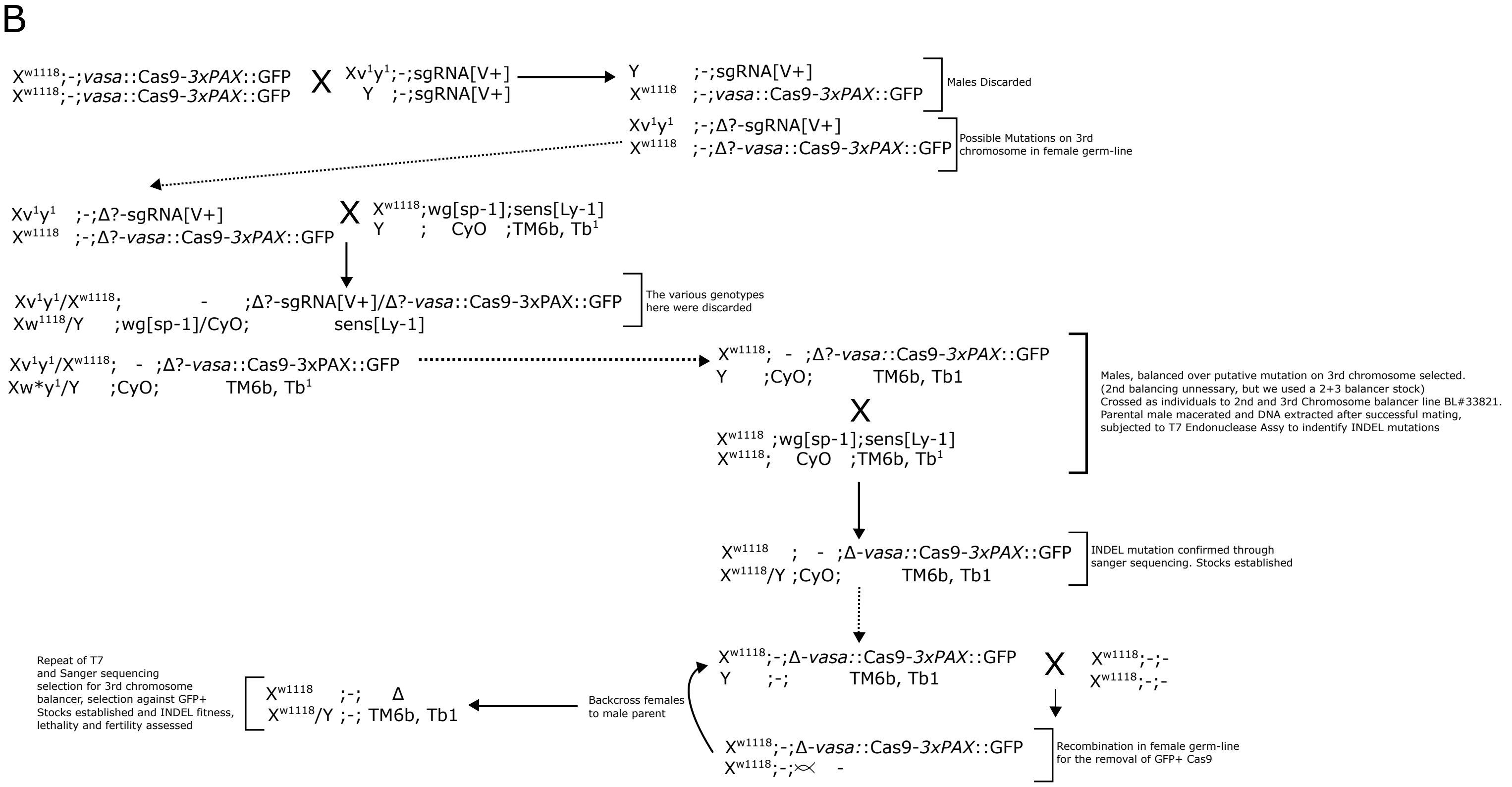

Supplementary Technical Cross 5: Synthetic Lethal Element Screen

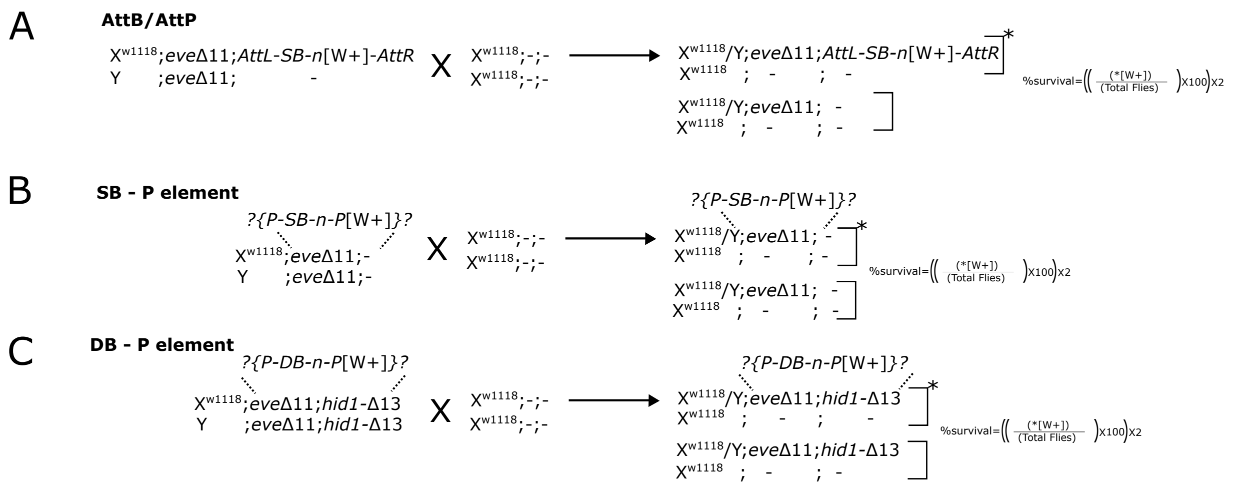

Supplementary Technical Cross 6: qRT-PCR Embryo Analysis for P-Element Insertions

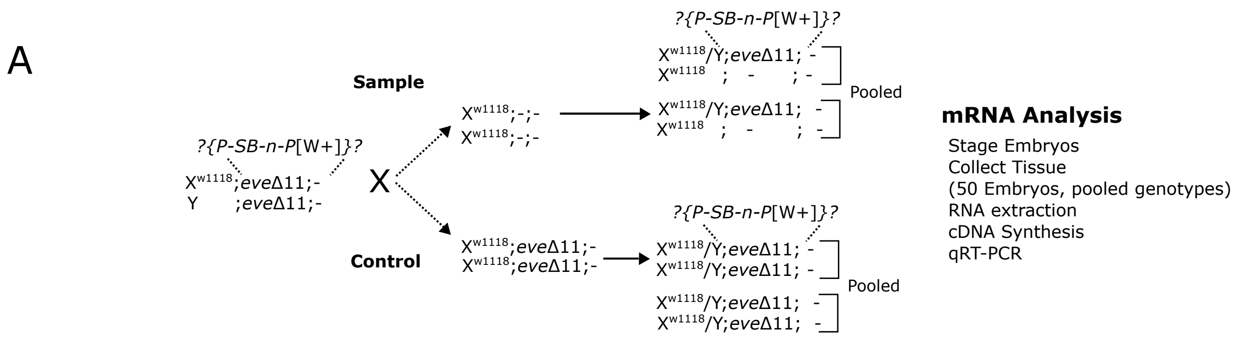

Supplement: Supplementary file 1 — Supplementary Information [file 41598_2018_31433_MOESM1_ESM.pdf]
